# The Geographical Differences in the Morphology of *Diptychus maculatus:* Environmental Driving Factors and Adaptive Evolution

**DOI:** 10.1002/ece3.72616

**Published:** 2026-01-12

**Authors:** Yichao Hao, Huimin Hao, Zhengwei Wang, Yinsheng Chen, Huale Lu, Jie Wei, Zhulan Nie

**Affiliations:** ^1^ College of Life Science and Technology Tarim University Alar China; ^2^ Xinjiang Production & Construction Corps Key Laboratory of Protection and Utilization of Biological Resources in Tarim Basin Alar China

**Keywords:** adaptive evolution, *D. maculatus*, environmental drivers, geographical populations, geometric morphometrics, morphological differentiation

## Abstract

As a typical cold‐water fish in the Schizothoracinae subfamily, *Diptychus maculatus* serves as an important model for understanding species adaptation through morphological differentiation and geographical environment interactions. This study characterized five geographical populations (Kizil River, Toshkan River, Muzati River, Karasu River, and Kyzyl River) in the Tarim River Basin. By integrating traditional morphology (12 traits measured with vernier calipers) and geometric morphometrics (21 landmarks), and using nonparametric tests, principal component analysis (PCA), cluster analysis (CA), and discriminant analysis (DA), we analyzed the environmental driving mechanisms of morphological variation. Results showed 34 morphological traits differed significantly (*p* < 0.01) among populations. The Kyzyl River population exhibited larger body size and expanded mouth cleft morphology, putatively adaptive to high altitude, low temperature, and food‐scarce feeding strategies. The Muzati River population showed greater caudal peduncle height and posterior dorsal fin origin, reflecting locomotor adaptation to rapid‐current habitats. The Kizil River population had reduced eye diameter, possibly linked to decreased visual dependence in reservoir slow‐flow environments. PCA revealed the first three components explained 87.3% of variance, reflecting body energy, cranial feeding, and sensory function modules. CA divided populations into two clades: Karasu‐Toshkan convergence due to moderate‐flow habitats, and Kizil River isolation. DA achieved 90.6% accuracy, identifying 19 key traits (e.g., interorbital distance, caudal peduncle height). This study reveals environmental and human‐induced (e.g., reservoir blockage) morphological adaptation, providing insights for plateau cold‐water fish research.

## Graphical abstract

This study morphologically analyzed 385 Diptychus maculatus from 5 Tarim River Basin populations. Using traditional and geometric morphometrics, it revealed environmental (high‐altitude cold, rapids, reservoir flows) and human‐driven morphological differentiation. 34 traits varied significantly, PCA explained 87.3% of variation, discriminant analysis achieved 90.6% accuracy, aiding plateau cold‐water fish adaptive evolution research and conservation.

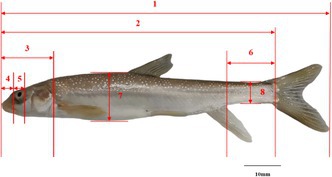

## 1 Introduction

*Diptychus maculatus* (the spotted thick‐lipped fish) belongs to the genus *Diptychus* of the family Cyprinidae within the order Cypriniformes. As a representative cold‐water species in the subfamily Schizothoracinae, it is primarily distributed in the Tarim River Basin and Ili River Basin of Xinjiang, China (Guo 2012). Serving as a typical example of adaptive evolution in plateau fishes, the long‐term interaction between its morphological traits and the complex geographical environment of its habitat renders it an ideal model for understanding the environmental adaptation and phenotypic divergence of species (Li et al. 2016). Phenotypic divergence in fishes driven by environmental factors constitutes a universal pattern. For instance, the divergence in growth strategies between lacustrine and riverine populations of African cichlids underscores the role of adaptive plasticity (Rajkov et al. 2018), whereas *Melanotaenia australis* exhibits plastic responses in body shape across water bodies with varying flow velocities (Kelley et al. 2017). However, for *D. maculatus* in the Tarim River Basin, in‐depth research remains lacking regarding whether different geographical populations within the same basin have evolved similar phenotypic divergence patterns in response to heterogeneous environmental pressures.

Owing to differences in hydrological conditions, food resources, and the intensity of human disturbances, the main stream and tributaries of the Tarim River Basin have formed multiple relatively isolated geographical units (Ge et al. 2024)—these serve as a natural experimental system for investigating environmentally driven phenotypic divergence. For instance, the Kyzyl River ecosystem is fragile and characterized by low productivity (Cui et al. 2019), which may drive adaptive changes in the feeding‐associated morphology of its populations; by contrast, the Muzati River exhibits unstable runoff and high sediment load (Zhang 2024; Zhao et al. 2020), which may select for individuals with a more streamlined body morphology. Although prior studies have documented the morphology of this species (Li 2023; Wang et al. 2024), most were confined to macroscopic descriptions and failed to effectively incorporate modern morphometric approaches. Consequently, it has been challenging to uncover fine‐scale phenotypic divergence at the population level and its adaptive significance—yet this is critical for evaluating the species' evolutionary potential and capacity to cope with environmental changes (Yin et al. 2024).

Furthermore, *D maculatus* is confronting severe threats to its survival. Hydrological perturbations driven by climate change have contracted its suitable habitats, whereas anthropogenic stressors—including water conservancy projects, overfishing, and pollution—have directly impaired its habitats and population structure (Bao et al. 2022; Bo et al. 2023). These pressures have resulted in a drastic decline in its population size, and the species was designated as a National Second‐Class Protected Animal in 2021 (Nie et al. 2025). Against this backdrop, gaining an in‐depth understanding of the phenotypic variation patterns and adaptive potential across its distinct populations is pivotal for developing targeted conservation strategies.

Given the limitations of traditional morphology in detecting fine‐scale phenotypic variation, such studies often fail to accurately capture subtle spatial variations in morphological structures—thus resulting in constrained precision of phenotypic analysis (Sharker et al. 2018; Wang et al. 2025; Zhao et al. 2023). This study will integrate traditional morphometrics with geometric morphometrics; the latter allows for precise quantification of morphological structures through anatomical landmarks, thereby substantially enhancing the precision of phenotypic analysis (Strauss and Bookstein 1982). This study focuses on *D*. *maculatus* populations from five rivers within the Tarim River Basin (the Kyzyl River, Toxkan River, Muzati River, Karasu River, and Kizil River) as research subjects, with the following objectives: (1) to quantify the significance of differences in morphological traits among distinct geographical populations; (2) to identify key morphological indices that drive population divergence; (3) to resolve population morphological clustering patterns and develop discriminant functions. The findings of this study will provide a refined morphological basis for the identification of *D. maculatus* populations. More importantly, by elucidating how environmental pressures shape its phenotype, this study will yield key insights into the species' evolutionary adaptation strategies in high‐altitude regions and offer scientific support for assessing the vulnerability of different populations, defining priority conservation units, and guiding the precise restoration of habitats.

## 2 Materials and Methods

### 2.1 Sample Collection

**FIGURE 1:**
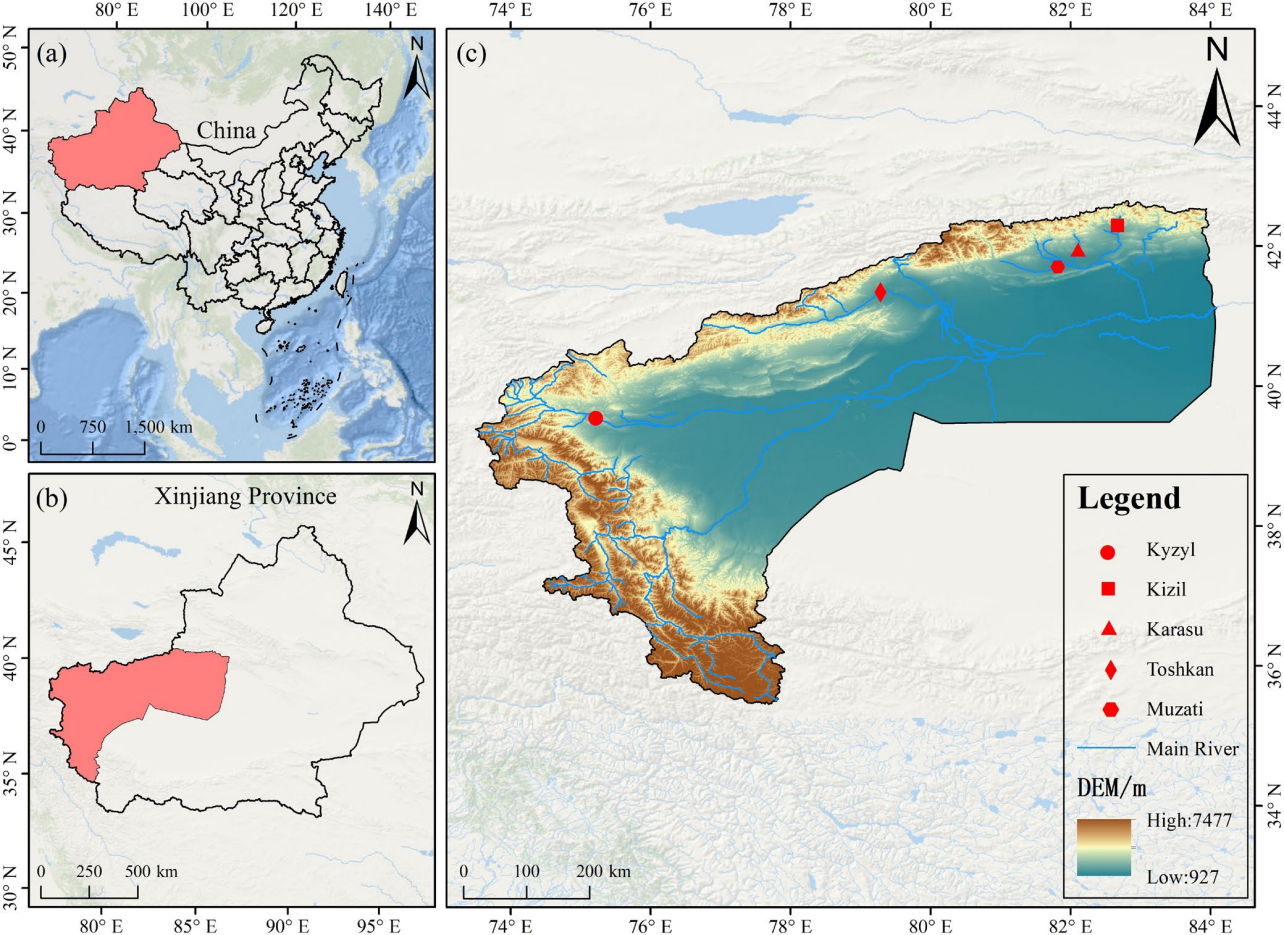
Diagram of sampling points.

In this study, five geographical populations were selected as research subjects in the Tarim River Basin, Xinjiang, namely the Kyzyl River, Toshkan River, Muzati River, Karasu River, and Kizil River. These rivers represent a typical hydrological gradient and habitat type variation within the basin, encompassing environments of high elevation, low temperature, and rapid current, mid‐reach slow current, as well as slow current and lentic water regulated by reservoirs. They exhibit good ecological representativeness, with sampling sites illustrated in Figure 1. The geographical coordinates, elevation, water temperature, and other parameters of each sampling site are provided in Table 1. These five rivers were chosen due to their high habitat heterogeneity, which can effectively reflect the potential for morphological adaptive divergence of *D. maculatus* under different environmental pressures.

**TABLE 1:** sampling site information.

Populations	Longitude and latitude	Altitude (m)	Water temperature (°C)
Muzati River	81°50′59″E, 41°37′20″N	1645	11.2
Kyzyl River	75°15′22″E, 39°43′13″N	2304	8.9
Karasu River	82°6′35″E, 41°57′0″N	1372	14.7
Toshkan River	79°17′24″E, 41°24′59″N	1372	15.2
Kizil River	82°38′3″E, 42°17′37″N	1542	14.5

From November 2023 to June 2024, sampling was conducted using cage nets (frame dimensions 25 × 20 cm, mesh size 4 mm). All sampling was conducted during daytime (09:00–17:00). The fish were anesthetized with MS‐222 (Fujian Jinjiang Aquatic Products Co. Ltd.) prior to on‐site measurements of their traditional morphological indices and geometric morphometric parameters, as specified in Table 2. All individuals were revived and returned to their original collection waters after measurement to ensure population conservation and ecological sustainability. All experimental procedures adhered to the Regulations for the Administration of Laboratory Animals in China and were approved by the Ethics Committee of Tarim University (approval number: PB20250627002).

**TABLE 2:** Sample information of *Diptychus maculatus*.

Populations	Sampling time	Number	Body weight (g)		Body length (mm)	
			Range	Mean ± SD	Range	Mean ± SD
Muzati River	2023.11	202	4.77–38.24	13.52 ± 6.46	68.49 ~ 152.29	106.23 ± 17.61
Kyzyl River	2023.12	55	8.72–9.26	16.82 ± 5.48	92.32 ~ 155.61	120.24 ± 14.21
Karasu River	2024.06	25	4.62–14.56	8.25 ± 2.43	70.53 ~ 105.12	86.25 ± 9.22
Toshkan River	2024.06	72	2.89–21.63	8.66 ± 3.78	55.41 ~ 120.87	88.22 ± 12.09
Kizil River	2024.06	31	1.10–3.50	1.96 ± 0.69	40.52 ~ 65.99	51.73 ± 6.35

### 2.2 Biological Measurements

**FIGURE 2:**
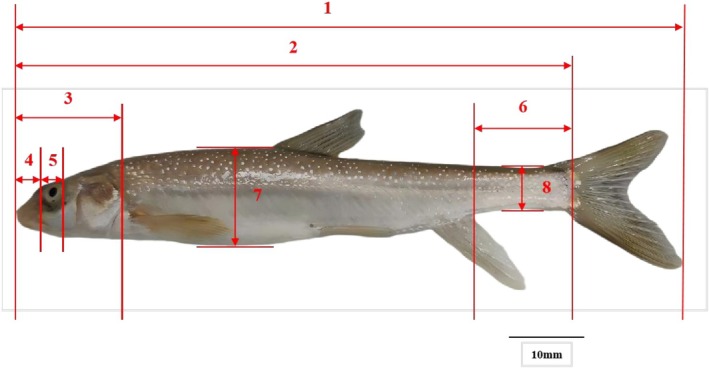
Traditional morphological measurement of *Diptychus maculatus*. 1. total length; 2. body length; 3. head length; 4. snout length; 5. eye diameter; 6. caudal peduncle length; 7. body depth; 8. caudal peduncle height.

After the experimental fish were anesthetized with MS‐222, their body surface moisture was wiped dry with a soft cloth. Body weight was measured using an electronic balance (precision: 0.01 g) and recorded as X13. Twelve traditional morphological traits (Figure 2) were measured with a digital vernier caliper (precision: 0.01 mm) and designated as X1, X2, X3, X4, X5, X6, X7, X8, X9, X10, X11, and X12. Measurement protocols followed (Wang et al. 2023), with details provided in Table 3.

**TABLE 3:** Descriptions of body weight and traditional traits of *Diptychus maculatus*.

Parameter	Traits	Parameter	Traits
*X*_1_	Total length	*X*_7_	Eye diameter
*X*_2_	Body length	*X*_8_	Interorbital distance
*X*_3_	Body width	*X*_9_	Mouth cleft wide
*X*_4_	Body depth	*X*_10_	Mouth cleft high
*X*_5_	Head length	*X*_11_	Caudal peduncle length
*X*_6_	Snout length	*X*_12_	Caudal peduncle height
*X*_13_	Body weight		

**FIGURE 3:**
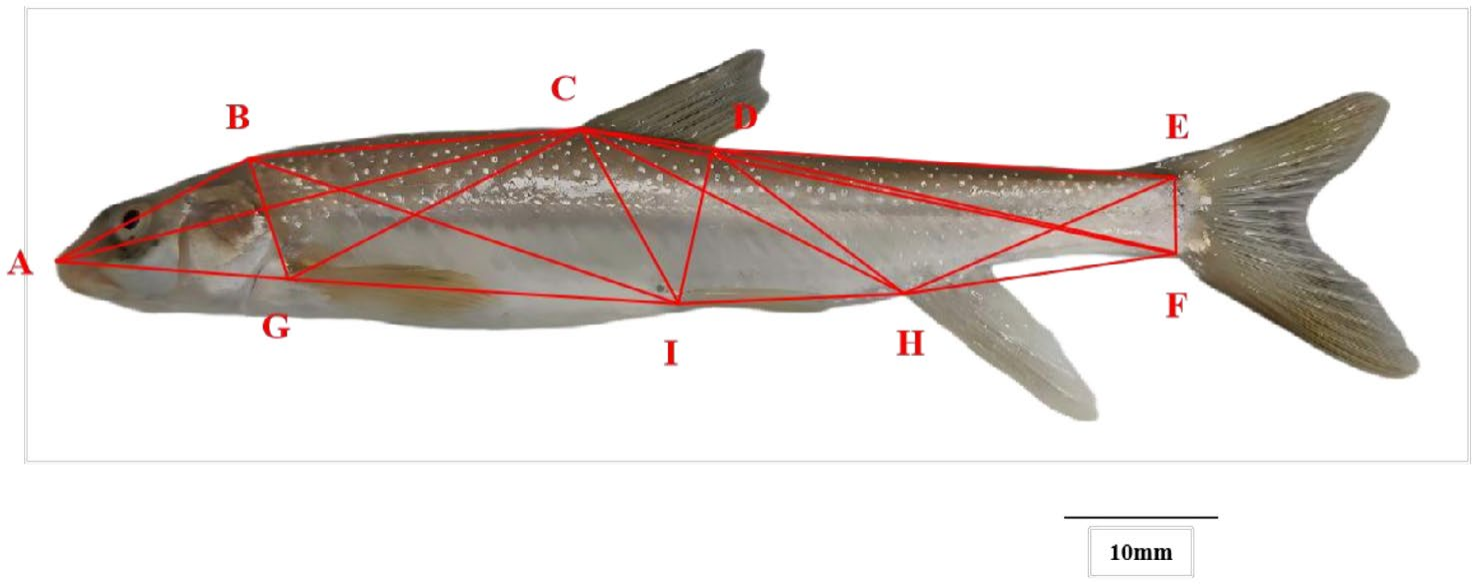
Frame measurement of *Diptychus maculatus*. (A) Tip of snout; (B) The end of the back of the head; (C) Origin of the dorsal fin; (D) Basal end of the dorsal fin; (E) Dorsal origin of the caudal fin; (F) Ventral origin of the caudal fin; (H) Anal fin origin; (I) Ventral fin origin; (J) Pectoral fin origin.

For geometric morphometric analysis, the two‐dimensional landmark method was employed. A Canon EOS 90D camera was fixed in place to capture lateral (left‐side) images of each fish, with a standardized scale bar applied. Nine landmarks were annotated using TPSDig2 software (v.2.32) (Figure 3): snout tip (A), posterior end of the dorsal cranial region (B), dorsal fin origin (C), posterior end of the dorsal fin base (D), dorsal origin of the caudal fin (E), ventral origin of the caudal fin (F), anal fin origin (H), pelvic fin origin (I), and pectoral fin origin (J). Based on these landmarks, 21 truss network traits were constructed, corresponding to the straight‐line distances between each pair of landmarks (X14–X34); details are presented in Table 4. This method effectively captures localized morphological variations and minimizes measurement errors (Strauss and Bookstein 1982).

**TABLE 4:** Descriptions of frame morphological traits of *Diptychus maculatus*.

Parameter	Traits	Description
*X*_14_	AB	The distance from tip of snout to the end of the back of the head
*X*_15_	BC	The distance from the end of the back of the head to origin of dorsal fin
*X*_16_	CD	The distance of dorsal fin (from origin to the end)
*X*_17_	DE	The distance from basal end of the dorsal fin to dorsal origin of caudal fin
*X*_18_	EF	The distance from dorsal origin of caudal fin to ventral origin of the caudal fin
*X*_19_	FH	The distance from ventral origin of the caudal fin to anal fin origin
*X*_20_	HI	The distance from anal fin origin to ventral fin origin
*X*_21_	IJ	The distance from ventral fin origin to pectoral fin origin
*X*_22_	AJ	The distance from tip of snout to pectoral fin origin
*X*_23_	AC	The distance from tip of snout to origin of dorsal fin
*X*_24_	AI	The distance from tip of snout to ventral fin origin
*X*_25_	BJ	The distance from the end of the back of the head to pectoral fin origin
*X*_26_	BI	The distance from the end of the back of the head to ventral fin origin
*X*_27_	CJ	The distance from origin of dorsal fin to pectoral fin origin
*X*_28_	CI	The distance from origin of dorsal fin to ventral fin origin
*X*_29_	CH	The distance from origin of dorsal fin to anal fin origin
*X*_30_	CF	The distance from origin of dorsal fin to ventral origin of the caudal fin
*X*_31_	DI	The distance from basal end of the dorsal fin to ventral fin origin
*X*_32_	DH	The distance from basal end of the dorsal fin to anal fin origin
*X*_33_	DF	The distance from basal end of the dorsal fin to ventral origin of the caudal fin
*X*_34_	EH	The distance from dorsal origin of caudal fin to anal fin origin

To account for the confounding effect of body size on morphological data, all morphological traits underwent logarithmic transformation (log_10_(x + 1)), followed by allometric analysis (ANCOVA, with body length as the covariate). The results revealed that most traits were significantly correlated with body length (*p* < 0.05); thus, residual analysis was utilized to extract size‐independent shape variables for subsequent analyses. Geometric morphometric data were further subjected to Procrustes superimposition to remove the effects of position, orientation, and scale, yielding Procrustes coordinates for statistical modeling.

### 2.3 Data Analysis

In this study, multiple statistical methods were employed to systematically analyze the morphological differences among five geographical populations of *D. maculatus*. All analyses were based on size‐corrected data following logarithmic transformation (unless otherwise specified), with the aims of eliminating the influence of individual body size on morphological traits and verifying whether morphological variation is independent of body size.

For geometric morphometric data, prior to the aforementioned multivariate analyses, Procrustes superposition was applied to eliminate non‐shape variations among samples caused by translation, rotation, and scaling. This step extracted pure shape information (i.e., Procrustes coordinates) for subsequent statistical analyses. All statistical analyses were conducted using IBM SPSS Statistics 26 software and Origin 2021. Processes related to geometric morphometrics—including Procrustes superposition and morphological space visualization—were completed in MorphoJ software (v.1.07a), with the significance level set at *p* < 0.05.

#### 2.3.1 Nonparametric Tests

The data failed to conform to a normal distribution, as confirmed by the Shapiro–Wilk test (*p* < 0.05). Consequently, the nonparametric Kruskal–Wallis *H* test was used to conduct an overall comparison of the 34 morphological traits. In cases where significant differences were detected, the Dunn‐Bonferroni method was further applied for post hoc pairwise comparisons to identify the specific sources of these differences. This analysis was designed to verify whether significant morphological divergences existed among populations across different habitats.

#### 2.3.2 Principal Component Analysis

Principal component analysis (PCA) was employed to perform dimensionality reduction on the residuals of 34 morphological traits (following allometric correction with body length as the covariate). By extracting principal components, PCA summarizes the variation structure within the multivariate morphological space, with emphasis on interpreting biological functional modules (e.g., body shape module, feeding‐related module) represented by principal components with high contribution rates. The analysis was based on a correlation matrix; principal components with eigenvalues > 1 were retained, and the biological significance of each principal component was interpreted using the factor loading matrix.

#### 2.3.3 Cluster Analysis

To further clarify the morphological similarity and taxonomic relationships among geographical populations, hierarchical cluster analysis (CA) was performed using Ward's method based on the Euclidean distance matrix. This analysis aimed to objectively identify the natural grouping patterns of populations, which were visualized via a dendrogram. In this way, it illustrates the shaping effect of different habitat types on morphological traits.

#### 2.3.4 Discriminant Analysis

To establish an effective population discrimination model and screen for key discriminatory traits, stepwise discriminant analysis (DA) was employed, with Wilks' Lambda statistic as the screening criterion (entry: *F* ≥ 3.84; removal: *F* ≤ 2.71). This analysis aimed to select a subset of variables that contribute most significantly to distinguishing the five geographical populations from all morphological variables, construct Fisher's linear discriminant functions, and calculate the discriminant accuracy via jackknife validation to evaluate the classification performance of the established model.

## 3 Results

In this study, multiple statistical methods were employed to systematically analyze the morphological variation patterns of *D. maculatus* across five geographical populations in the Tarim River Basin. The results not only revealed prevalent morphological differences among populations but also identified key discriminatory traits and their potential correlations with habitat adaptation.

### 3.1 Results of Nonparametric Tests

Results of the Kruskal–Wallis *H* test indicated that all 34 measured morphological traits exhibited highly significant differences among the five geographical populations (*p* < 0.01 for all traits). This preliminarily confirms that geographical isolation and habitat heterogeneity have driven significant phenotypic divergence in *D*. *maculatus* (see Table 5 for details).

**TABLE 5:** Analysis of the Differences in Various Traits of *Diptychus maculatus* in Different Geographical Populations.

Traits	Muzati River	Kyzyl River	Karasu River	Toshkan River	Kizil River	*H*	*P*
*X*_1_	2.11 (2.05, 2.16)^b^	2.15 (2.12, 2.18)^a^	2.02 (1.97, 2.04)^c^	2.03 (1.99, 2.06)^c^	1.79 (1.74, 1.84)^d^	186.777	0.000
*X*_2_	2.02 (1.96, 2.07)^b^	2.08 (2.05, 2.11)^a^	1.94 (1.90, 1.96)^c^	1.94 (1.90, 1.97)^c^	1.71 (1.67, 1.75)^d^	189.285	0.000
*X*_3_	1.10 (1.03, 1.16)^a^	1.13 (1.06, 1.18)^a^	0.98 (0.95, 1.02)^b^	1.02 (0.96, 1.07)^b^	0.76 (0.70, 0.81)^d^	142.876	0.000
*X*_4_	1.19 (1.15, 1.25)^a^	1.22 (1.17, 1.27)^a^	1.06 (1.03, 1.12)^bd^	1.13 (1.07, 1.13)^b^	0.87 (0.83, 0.94)^d^	167.373	0.000
*X*_5_	1.34 (1.28, 1.40)^b^	1.41 (1.38, 1.44)^a^	1.31 (1.27, 1.33)^bc^	1.28 (1.25, 1.32)^c^	1.10 (1.06, 1.14)^d^	165.605	0.000
*X*_6_	0.84 (0.76, 0.92)^b^	0.95 (0.91, 0.99)^a^	0.80 (0.76, 0.85)^bc^	0.78 (0.73, 0.82)^c^	0.56 (0.49, 0.61)^d^	160.930	0.000
*X*_7_	0.67 (0.62, 0.70)^ab^	0.69 (0.64, 0.72)^a^	0.64 (0.61, 0.67)^b^	0.65 (0.61, 0.69)^b^	0.52 (0.48, 0.55)^d^	75.468	0.000
*X*_8_	0.84 (0.78, 0.90)^b^	0.96 (0.91, 0.98)^a^	0.65 (0.60, 0.72)^cd^	0.71 (0.66, 0.77)^c^	0.51 (0.40, 0.59)^d^	232.138	0.000
*X*_9_	0.86 (0.77, 0.92)^b^	0.96 (0.92, 0.98)^a^	0.84 (0.79, 0.90)^bc^	0.79 (0.75, 0.88)^c^	0.62 (0.55, 0.69)^d^	142.592	0.000
*X*_10_	0.79 (0.70, 0.87)^b^	0.91 (0.85, 0.95)^a^	0.85 (0.82, 0.88)^ab^	0.81 (0.77, 0.85)^b^	0.56 (0.49, 0.67)^c^	120.579	0.000
*X*_11_	1.29 (1.24, 1.35)^b^	1.36 (1.31, 1.39)^a^	1.26 (1.23, 1.30)^bc^	1.18 (1.14, 1.26)^c^	1.05 (0.90, 1.08)^d^	166.881	0.000
*X*_12_	0.89 (0.83, 0.94)^b^	0.94 (0.91, 0.99)^a^	0.81 (0.77, 0.85)^c^	0.77 (0.74, 0.82)^c^	0.60 (0.55, 0.66)^d^	195.081	0.000
*X*_13_	1.09 (0.92, 1.23)^b^	1.20 (1.15, 1.27)^a^	0.91 (0.79, 0.98)^c^	0.90 (0.79, 0.99)^c^	0.25 (0.15, 0.41)^d^	161.823	0.000
*X*_14_	1.27 (1.21, 1.32)^b^	1.32 (1.28, 1.36)^a^	1.23 (1.18, 1.27)^c^	1.21 (1.17, 1.24)^c^	1.00 (0.97, 1.04)^d^	146.532	0.000
*X*_15_	1.48 (1.41, 1.54)^b^	1.54 (1.50, 1.58)^a^	1.31 (1.27, 1.38)^cd^	1.34 (1.29, 1.39)^c^	1.15 (1.10, 1.19)^d^	214.087	0.000
*X*_16_	1.13 (1.06, 120)^b^	1.18 (1.12, 1.21)^a^	1.09 (1.03, 1.15)^b^	1.13 (1.08, 1.17)^b^	0.80 (0.78, 0.85)^c^	104.071	0.000
*X*_17_	1.64 (1.57, 1.71)^b^	1.70 (1.66,1.74)^a^	1.51 (1.47, 1.58)^c^	1.51 (1.47, 1.55)^c^	1.29 (1.24, 1.36)^d^	203.361	0.000
*X*_18_	0.93 (0.87, 0.99)^b^	0.97 (0.93,1.02)^a^	0.85 (0.82, 0.88)^c^	0.82 (0.80, 0.87)^c^	0.59 (0.53, 0.65)^d^	169.603	0.000
*X*_19_	1.45 (1.39, 1.50)^b^	1.51 (1.47,1.54)^a^	1.29 (1.23, 1.35)^cd^	1.36 (1.32, 1.39)^c^	1.07 (1.04, 1.16)^d^	182.422	0.000
*X*_20_	1.36 (1.28, 1.42)^a^	1.39 (1.34, 1.43)^a^	1.17 (1.13, 1.25)^bc^	1.22 (1.17, 1.26)^b^	0.98 (0.89, 1.06)^c^	208.592	0.000
*X*_21_	1.51 (1.45, 1.57)^b^	1.59 (1.53, 1.61)^a^	1.42 (1.38, 1.45)^c^	1.44 (1.41, 1.48)^c^	1.15 (1.11, 1.22)^d^	170.020	0.000
*X*_22_	1.38 (1.33, 143)^b^	1.44 (1.38, 1.46)^a^	1.30 (1.24, 1.34)^c^	1.29 (1.25, 1.31)^c^	1.10 (1.07, 1.13)^d^	195.013	0.000
*X*_23_	1.68 (1.62, 1.73)^b^	1.74 (1.70, 1.75)^a^	1.57 (1.52, 1.59)^c^	1.57 (1.53, 1.61)^c^	1.37 (1.34, 1.42)^d^	205.833	0.000
*X*_24_	1.74 (1.69, 1.79)^b^	1.81 (1.77, 1.83)^a^	1.66 (1.62, 1.69)^c^	1.67 (1.63, 1.70)^c^	1.44 (1.40, 1.48)^d^	186.876	0.000
*X*_25_	1.16 (1.11, 1.21)^a^	1.19 (1.16, 1.22)^a^	1.07 (1.04, 1.14)^b^	1.10 (1.49, 1.54)^b^	0.90 (0.85, 0.94)^c^	140.793	0.000
*X*_26_	1.61 (1.55, 1.66)^b^	1.67 (1.65, 1.71)^a^	1.50 (1.44, 1.56)^cd^	1.52 (1.49, 1.54)^c^	1.30 (1.27, 1.37)^d^	194.815	0.000
*X*_27_	1.43 (1.38, 1.48)^b^	1.50 (1.46, 1.53)^a^	1.32 (1.25, 1.35)^c^	1.32 (1.29, 1.38)^c^	1.11 (1.06, 1.19)^d^	193.719	0.000
*X*_28_	1.25 (1.20, 1.30)^a^	1.29 (1.24, 1.32)^a^	1.18 (1.11, 1.22)^b^	1.20 (1.16, 1.24)^b^	0.93 (0.87, 0.99)^c^	134.001	0.000
*X*_29_	1.52 (1.46, 1.59)^b^	1.58 (1.54, 1.61)^a^	1.44 (1.38, 1.46)^c^	1.44 (1.40, 1.49)^c^	1.21 (1.17, 1.27)^d^	172.757	0.000
*X*_30_	1.77 (1.71, 1.83)^b^	1.83 (1.78, 1.86)^a^	1.67 (1.62, 1.71)^c^	1.69 (1.65, 1.71)^c^	1.43 (1.38, 1.48)^d^	180.028	0.000
*X*_31_	1.16 (1.11, 1.22)^a^	1.17 (1.15, 1.22)^a^	1.05 (1.00, 1.11)^b^	1.12 (1.06, 1.15)^b^	0.83 (0.77, 0.86)^c^	143.614	0.000
*X*_32_	1.34 (1.28, 1.41)^b^	1.40 (1.35, 1.46)^a^	1.23 (1.19, 1.28)^c^	1.19 (1.13, 125)^c^	0.99 (0.96, 1.09)^d^	208.873	0.000
*X*_33_	1.65 (1.59, 1.71)^b^	1.71 (1.67, 1.75)^a^	1.55 (1.52, 1.60)^c^	1.53 (1.49, 1.57)^c^	1.29 (1.25, 1.36)^d^	203.421	0.000
*X*_34_	1.45 (1.40, 1.52)^b^	1.54 (1.48, 1.56)^a^	1.34 (1.30, 1.39)^c^	1.37 (1.34, 1.41)^c^	1.11 (1.06, 1.17)^d^	189.893	0.000

*Note:* Different superscripts in the same row indicate significant differences among populations (*P* < 0.05); All values in the table represent the median (lower quartile, upper quartile), example: 2.11 (2.05, 2.16).

However, the pervasiveness of significant differences underscores the necessity of conducting multiple comparison tests. Results of the Dunn‐Bonferroni post hoc test revealed no significant differences in the vast majority of traits between the Karasu River and Toshkan River populations (*p* > 0.05), indicating that these two populations are morphologically the most similar. In contrast, the Kizil River population displayed highly significant differences from all other populations, with generally the smallest mean values across all morphological indices (see Table 5), reflecting its unique morphological characteristics. Specifically, the Kyzyl River population was significantly larger than other populations in terms of overall body size (e.g., body length X_2_, body weight X_13_) and oral structures (e.g., mouth gape width X_9_, mouth gape height X_10_); whereas the Muzati River population showed prominence in traits associated with swimming ability (e.g., caudal peduncle height X_12_, distance from dorsal fin origin to ventral origin of the caudal fin X_30_). The direction and magnitude of these differences provide a foundation for subsequent analyses of environment‐driven mechanisms.

### 3.2 Results of Principal Component Analysis

Principal component analysis (PCA) effectively reduced the dimensionality of the 34 morphological traits. The cumulative contribution rate of the first three principal components (PCs) reached 87.30%, which adequately represented the variation information of the original data (Table 6). The KMO test value (0.987) and Bartlett's test of sphericity (*p* < 0.001) confirmed that the data was highly suitable for PCA.

**TABLE 6:** PCA of the Traits of the *Diptychus maculatus* in Five Geographical Populations.

Traits	Principal component		
	PC1	PC2	PC3
*X*_1_	0.984	−0.025	−0.004
*X*_24_	0.983	−0.025	−0.018
*X*_2_	0.983	−0.026	−0.012
*X*_13_	0.983	−0.014	0.003
*X*_23_	0.976	−0.081	−0.024
*X*_21_	0.960	0.013	−0.006
*X*_29_	0.958	−0.032	−0.014
*X*_30_	0.955	−0.059	−0.041
*X*_17_	0.946	−0.113	−0.102
*X*_22_	0.945	−0.05	−0.047
*X*_27_	0.942	−0.055	−0.011
*X*_33_	0.940	−0.11	−0.083
*X*_26_	0.940	−0.056	−0.038
*X*_15_	0.939	−0.112	−0.035
*X*_3_	0.934	−0.037	0.059
*X*_14_	0.934	0.068	0.002
*X*_5_	0.931	0.098	0.003
*X*_34_	0.926	0.000	−0.104
*X*_19_	0.923	−0.044	−0.068
*X*_4_	0.923	−0.091	0.079
*X*_20_	0.922	−0.172	0.044
*X*_28_	0.911	0.001	0.06
*X*_8_	0.907	−0.101	0.079
*X*_25_	0.896	0.036	−0.019
*X*_32_	0.895	−0.191	−0.037
*X*_11_	0.888	0.06	−0.073
*X*_31_	0.888	−0.061	0.136
*X*_12_	0.882	0.03	−0.026
*X*_18_	0.872	−0.099	0.059
*X*_9_	0.834	0.291	−0.173
*X*_6_	0.825	0.203	0.188
*X*_16_	0.824	0.131	0.129
*X*_7_	0.614	0.423	0.588
*X*_10_	0.606	0.637	−0.4
Eigenvalue	28.094	0.909	0.679
Contribution rate	82.63	2.67	2.00
Cumulative contribution rate	82.63	85.30	87.30

**FIGURE 4:**
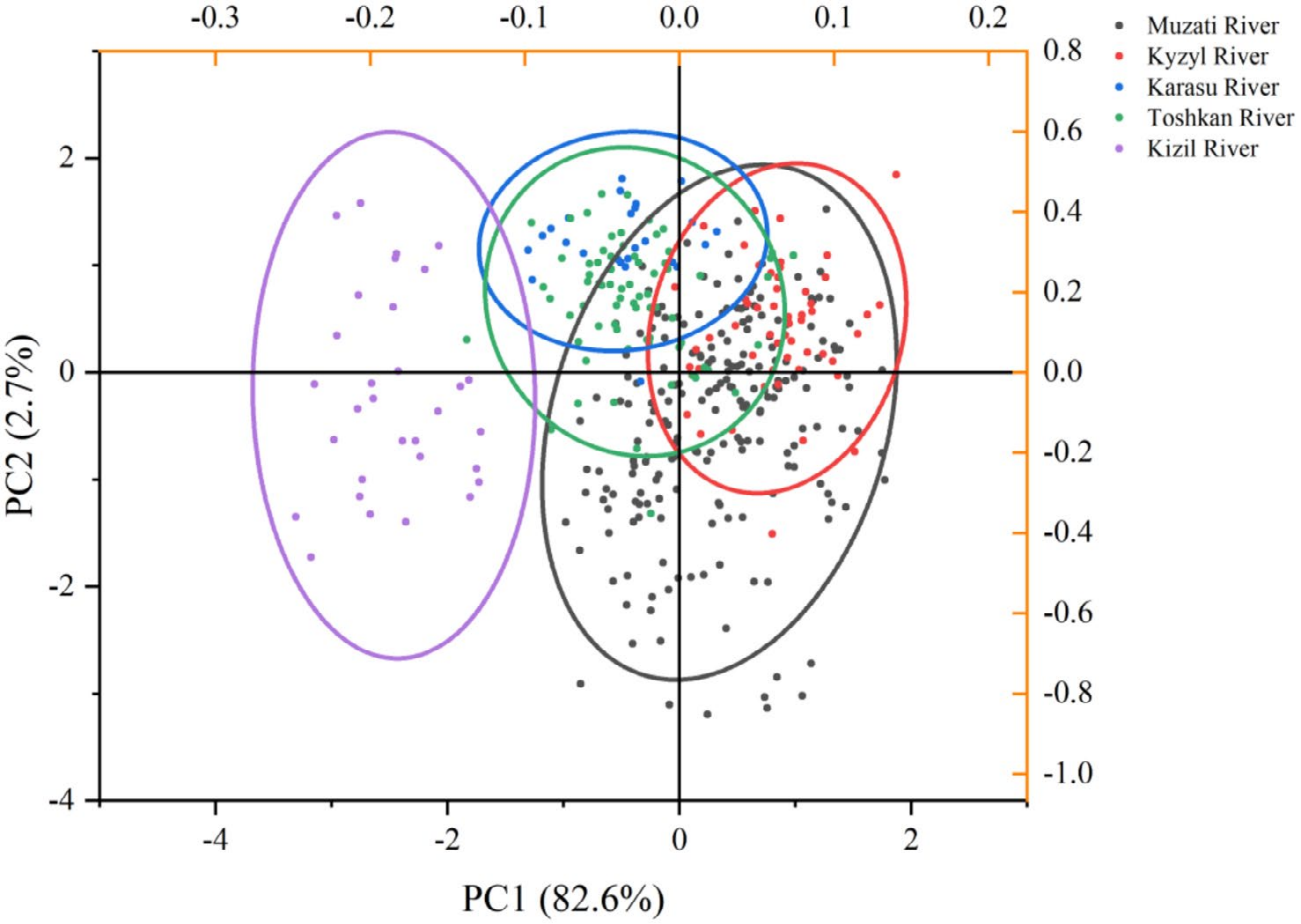
The distribution of five *Diptychus maculatus* populations on PC 1 and PC 2.

PC1 (contribution rate: 82.63%) served as the primary axis of morphological variation. Almost all traits (e.g., total length X_1_, body length X_2_, body weight X_13_, head length X_5_) exhibited high positive loadings (> 0.6) on PC1. This component primarily captured the comprehensive variation associated with overall body size and could be interpreted as the “body size‐energy module”. In the scatter plot (Figure 4), PC1 clearly discriminated the smaller‐bodied Kizil River population (concentrated on the left) from the larger‐bodied remaining populations (distributed on the right).

**FIGURE 5:**
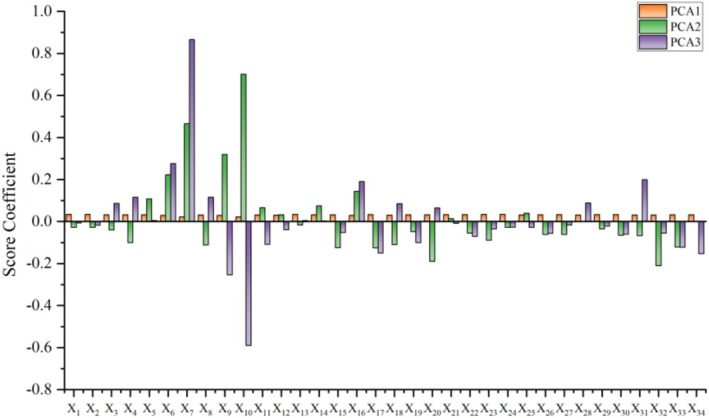
PCA1, PCA2, and PCA3 score coefficients.

PC2 (contribution rate: 2.67%) and PC3 (contribution rate: 2.00%) mostly reflected shape variations linked to specific functional traits. Traits with high loadings on PC2 included mouth gape height (X_10_, 0.637), eye diameter (X_7_, 0.423), and mouth gape width (X_9_, 0.291)—these primarily represented variations in the head's feeding and sensory organs. Traits with high loadings on PC3 were eye diameter (X_7_, 0.588) and snout length (X_6_, 0.188), further highlighting the differentiation of the head's sensory functions. As shown in the scatter plot (Figure 4), the populations from the Muzati River, Kyzyl River, Toshkan River, and Karasu River partially overlapped along the PC2 and PC3 axes but exhibited distinct trends, indicating subtle differences in their feeding and sensory strategies. In contrast, the Kizil River population was separated from all other populations across all these dimensions. The loading structure of the principal components is clearly depicted in the score coefficient plot (Figure 5). Among these components, numerous body size‐associated traits display high and consistent coefficients on PC1, whereas the key traits underlying PC2 and PC3 are distinctly emphasized.

### 3.3 Results of Cluster Analysis

**FIGURE 6:**
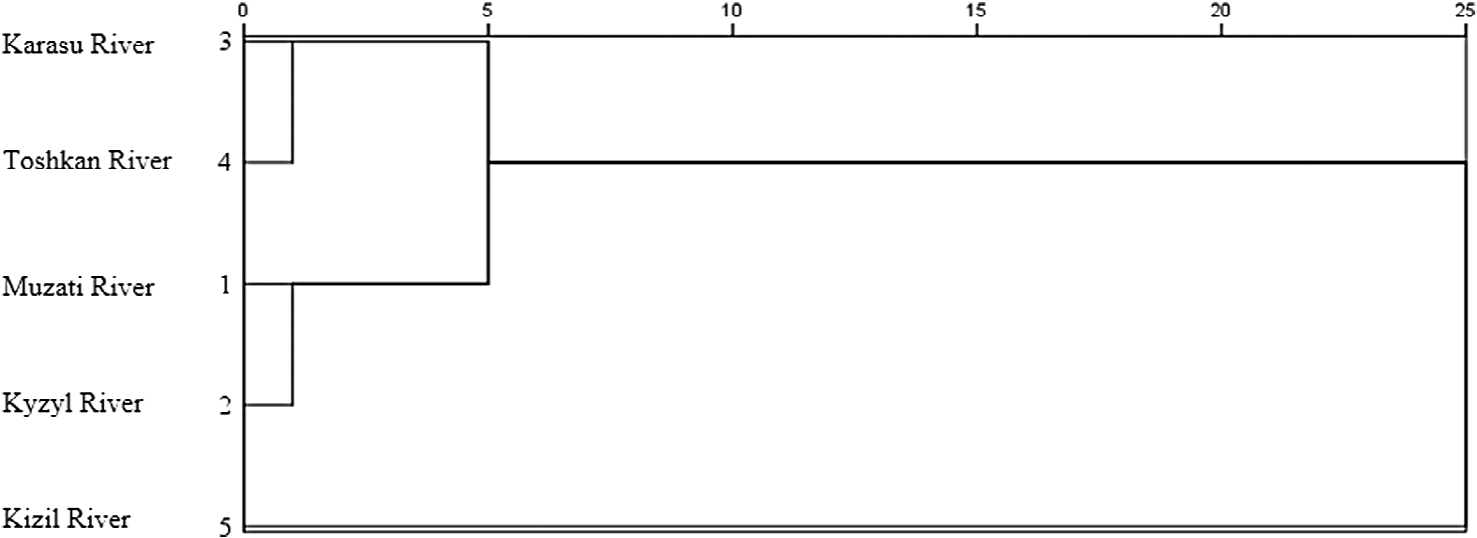
Cluster analysis of five geographical populations of *Diptychus maculatus*.

The results of hierarchical cluster analysis (Figure 6) were mutually corroborative with those of PCA and difference analysis. The squared Euclidean distance matrix (Table 7) revealed that the distance between the Karasu River and Toshkan River populations was the smallest (0.036), which supports the finding that these two populations exhibit high morphological similarity. In contrast, the Kizil River population showed the largest distances from all other populations (e.g., a distance of 5.340 from the Kyzyl River population), indicating that it has the most distinct morphological characteristics.

**TABLE 7:** The squared Euclidean distances among five geographical populations of *D*. *maculatus*.

Populations	Squared euclidean distance				
	Muzati River	Kyzyl River	Karasu River	Toshkan River	Kizil River
Muzati River	—	0.143	0.385	0.296	3.876
Kyzyl River		—	0.876	0.771	5.340
Karasu River			—	0.036	2.003
Toshkan River				—	2.156
Kizil River					—

The cluster dendrogram (Figure 6) partitioned the five populations into two major clades: the Kizil River population formed an independent clade, while the remaining four populations clustered into the other major clade. Within this latter clade, the Karasu River and Toshkan River populations clustered together first, followed by aggregation with the Muzati River and Kyzyl River populations. This clustering pattern clearly reflects morphological convergence among populations driven by habitat similarity.

### 3.4 Results of Discriminant Analysis

Stepwise discriminant analysis (DA) selected 19 variables that contributed most significantly to population discrimination from the 34 morphological traits (Table 8), and established discriminant functions for the five geographical populations. The Wilks' Lambda test confirmed that the established discriminant functions were of highly significant statistical significance (*P* < 0.001). The discriminant equations are presented as follows:

**TABLE 8:** Variable input process.

Steps	Variable input	Lambda							
		Statistics	df 1	df 2	df 3	*F*			
						Statistics	df 1	df 2	*p*
1	*X*_8_	0.389	1	4	380	149.005	4	380	0
2	*X*_1_	0.293	2	4	380	80.337	8	758	0
3	*X*_13_	0.208	3	4	380	67.656	12	1000.385	0
4	*X*_9_	0.158	4	4	380	59.635	16	1152.392	0
5	*X*_15_	0.13	5	4	380	53.018	20	1248.001	0
6	*X*_4_	0.105	6	4	380	49.534	24	1309.429	0
7	*X*_16_	0.092	7	4	380	45.189	28	1349.898	0
8	*X*_12_	0.08	8	4	380	42.379	32	1377.151	0
9	*X*_31_	0.072	9	4	380	39.443	36	1395.794	0
10	*X*_32_	0.065	10	4	380	37.072	40	1408.644	0
11	*X*_6_	0.06	11	4	380	34.861	44	1417.482	0
12	*X*_19_	0.057	12	4	380	32.658	48	1423.465	0
13	*X*_3_	0.054	13	4	380	30.867	52	1427.369	0
14	*X*_9_	0.051	14	4	380	29.368	56	1429.725	0
15	*X*_22_	0.048	15	4	380	28.045	60	1430.908	0
16	*X*_5_	0.045	16	4	380	26.992	64	1431.187	0
17	*X*_24_	0.043	17	4	380	25.973	68	1430.758	0
18	*X*_25_	0.041	18	4	380	24.966	72	1429.768	0
19	*X*_11_	0.039	19	4	380	24.07	76	1428.327	0

Muzati River Group:

$$\begin{array}{cc} Y= & -144.98+0.962X_{1}+0.047X_{3}+3.76X_{4}\\ & -0.63X_{5}+1.004X_{6}+0.168X_{8}-1.413X_{9}\\ & +0.288X_{10}-0.334X_{11}+3.594X_{12}-9.44X_{13}\\ & +1.651X_{15}-0.667X_{19}+0.518X_{22}+1.876X_{24}\\ & +1.07X_{25}+2.335X_{31}+1.038X_{32}. \end{array}$$

Kyzyl River Group:

$$\begin{array}{cc} Y= & -160.862+0.85X_{1}-0.751X_{3}+3.534X_{4}\\ & -0.433X_{5}+1.734X_{6}+2.044X_{8}-0.84X_{9}\\ & +1.089X_{10}-0.278X_{11}+3.669X_{12}-9.645X_{13}\\ & +1.777X_{15}+0.038X_{16}-0.662X_{19}+0.467X_{22}\\ & +2.173X_{24}+0.551X_{25}+2.072X_{31}+1.018X_{32}. \end{array}$$

Karasu River Group:

$$\begin{array}{cc} Y= & -114.716+0.873X_{1}+0.399X_{3}+2.687X_{4}\\ & +0.259X_{5}+1.295X_{6}-2.199X_{8}-0.369X_{9}\\ & +1.587X_{10}+0.130X_{11}+3.583X_{12}-8.075X_{13}\\ & +0.851X_{15}+0.516X_{16}-1.102X_{19}-0.015X_{22}\\ & +1.648X_{24}+0.995X_{25}+1.79X_{31}+0.943X_{32}. \end{array}$$

Toshkan River Group:

$$\begin{array}{cc} Y= & -119.918+0.899X_{1}+0.591X_{3}+3.337X_{4}\\ & -0.072X_{5}+0.854X_{6}-0.420X_{8}-1.048X_{9}\\ & +1.421X_{10}-0.438X_{11}+2.114X_{12}-8.550X_{13}\\ & +1.105X_{15}+0.637X_{16}-0.814X_{19}-0.18X_{22}\\ & +1.939X_{24}+1.107X_{25}+2.037X_{31}+0.621X_{32}. \end{array}$$

Kizil River Group:

$$\begin{array}{cc} Y= & -61.734+0.565X_{1}+0.008X_{3}+2.427X_{4}\\ & +0.178X_{5}+0.573X_{6}-0.344X_{8}-0.123X_{9}\\ & +0.362X_{10}-0.191X_{11}+2.508X_{12}-6.278X_{13}\\ & +0.982X_{15}-0.006X_{16}-0.696X_{19}+0.147X_{22}\\ & +1.373X_{24}+0.639X_{25}+1.212X_{31}+0.589X_{32}. \end{array}$$

**FIGURE 7:**
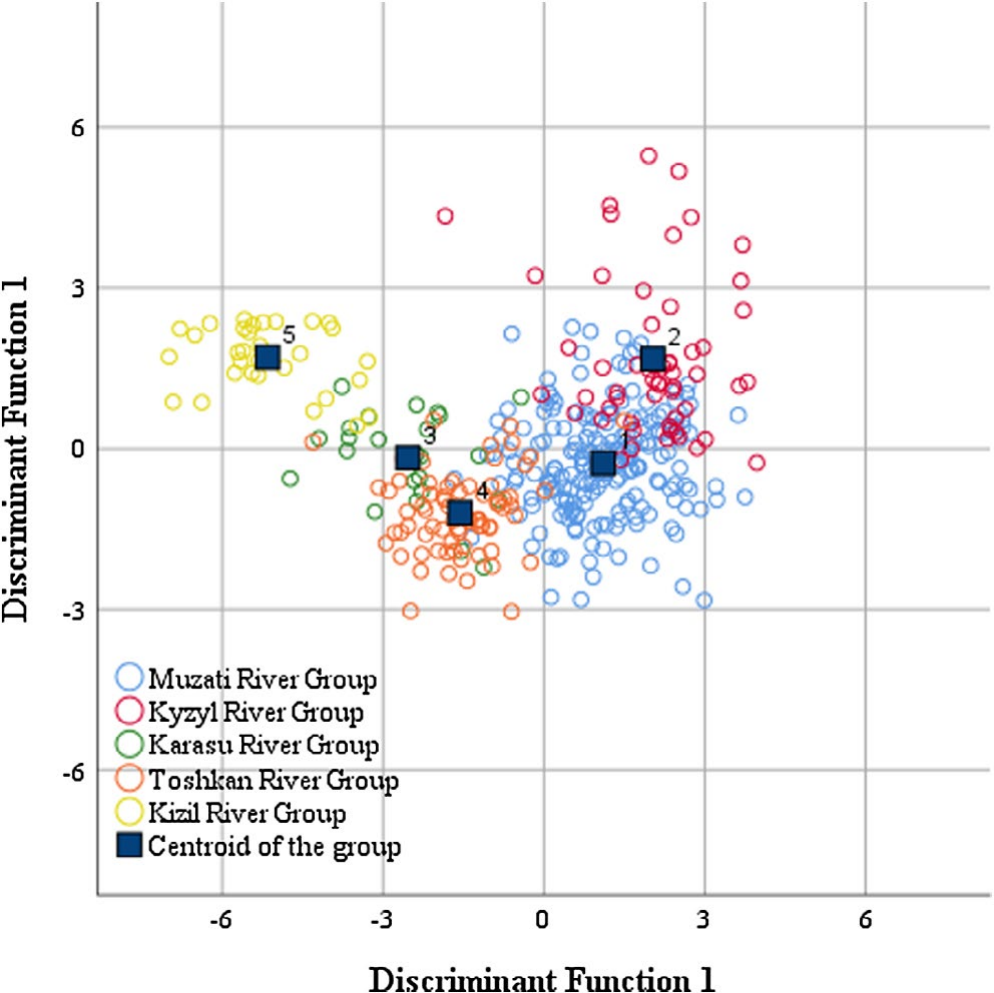
Discriminant analysis of morphological measurement characteristics of five geographical populations of *Diptychus maculatus*.

The scatter plot of discriminant functions (Figure 7) visually illustrates the distribution of the five populations within the discriminant space. The Karasu River and Toshkan River populations exhibited adjacent and partially overlapping distribution areas, which further confirms their morphological similarity; in contrast, the Kizil River population was completely segregated from all other populations.

Results of jackknife validation (Table 9) revealed that the overall discriminant accuracy of the discriminant functions was as high as 90.6%. Among the five populations, the discriminant accuracy for the Kizil River population reached 100%, while those for the Muzati River, Karasu River, and Toshkan River populations all exceeded 90%; the accuracy for the Kyzyl River population also reached 74.5%. These 19 key discriminatory traits (e.g., interorbital distance X_8_, caudal peduncle height X_12_) form an efficient morphological index system for distinguishing the geographical populations of *D. maculatus* in the Tarim River Basin.

**TABLE 9:** Identification results by discriminant function.

Population	Discriminant results					Number	Accuracy rate	Comprehensive discrimination rate
	Muzati River	Kyzyl River	Karasu River	Toshkan River	Kizil River			
Muzati River	189	9	0	4	0	202	93.60	90.60
Kyzyl River	14	41	0	0	0	55	74.50	
Karasu River	1	0	23	1	0	25	92.00	
Toshkan River	6	0	0	65	1	72	90.30	
Kizil River	0	0	0	0	31	31	100.00	

## 4 Discussion

### 4.1 Potential Driving Factors of Morphological Differences and Their Associations With the Environment

In this study, extensive and highly significant morphological differences were detected among the five geographical populations. Integrating the measured environmental data from the sampling sites (Table 1), we found that these patterns of morphological variation were highly spatially coupled with key environmental gradients, indicating a strong shaping effect of environmental filtering on phenotypes.

The Kyzyl River population (2304 m altitude, 8.9°C water temperature) exhibits the largest body size, consistent with the intraspecific pattern of “Bergmann's rule”—whereby cold environments tend to select for or shape larger body sizes. This likely relates to a survival strategy of extending the growth cycle and reducing metabolic rates to address energy constraints (Rypel 2014). Meanwhile, the significantly enlarged mouth gape structures (X_9_, X_10_) in this population probably represent an adaptive trait evolved to enhance foraging efficiency and offset insufficient energy intake in its relatively barren high‐altitude habitat (Guo et al. 2024; Combrink et al. 2023). However, it should be explicitly noted that the phenotypic differences observed in this study result from the combined effects of phenotypic plasticity (the capacity of individuals to adjust their morphology across different environments) and local adaptation (genetically differentiated evolution). Given the current data, we cannot precisely quantify the relative contributions of these two mechanisms, and future common garden experiments or genomic studies will help clarify the underlying mechanisms.

The Muzati River population (1645 m altitude, 11.2°C water temperature) exhibits locomotor morphological traits associated with fast‐flowing habitats, including a taller caudal peduncle (X_12_) and a more posterior dorsal fin origin (as reflected in truss traits such as X_15_). These traits are generally acknowledged to enhance swimming stability and efficiency in complex water currents (Binning and Roche 2015; Hetzel and Forsythe 2023). The population's shorter snout length (X_6_) may also correlate with its habit of benthic feeding in high‐sediment waters (López‐Fernández et al. 2014; Chen et al. 2021). While water velocity was not directly measured in this study, the known hydrological characteristics of the Muzati River (e.g., high flood peak discharge, unstable runoff) provide a credible ecological context for interpreting these morphological features.

The Kizil River population (sampling site adjacent to a reservoir, water temperature 14.5°C) is the most morphologically distinct, characterized by the smallest body size and a significant reduction in eye diameter (X_7_). This pattern is closely associated with the slow‐flowing, lentic environment shaped by reservoir regulation. Low‐flow environments reduce the requirement for sustained swimming performance, potentially inducing shifts in energy allocation strategies (Gilbert et al. 2020; Chai et al. 2023); meanwhile, alterations in light conditions (e.g., reduced transparency) that often accompany reservoir systems may also diminish the role of vision in foraging, thereby driving the reduction in eye diameter (Coghlan et al. 2024; Dgebuadze et al. 2020). The Kizil River population serves as a typical case of significant phenotypic divergence arising from the combined effects of geographical isolation (reservoir‐induced barrier) and environmental pressures.

### 4.2 Functional Module Analysis of Morphological Variation

The morphological variation modules identified via principal component analysis (PCA) reflect the integrated response strategies of *D maculatus* to distinct habitat pressures. PC1, defined as the “body size‐energy module” with a contribution rate exceeding 80%, demonstrates strong explanatory power—indicating that energy allocation strategies along the temperature‐altitude gradient are the primary driver of phenotypic divergence in *D. maculatus*. In contrast, the “head functional module” composed of PC2 and PC3, despite their relatively lower contribution rates, still uncovers more refined adaptive modifications linked to feeding niche differentiation at the local scale.

The most compelling evidence for environmental filtering in this study originates from the Karasu River and Toxkan River populations. Geographically, these two populations are not directly connected; however, they exhibit no significant differences in most of the 34 morphological traits, overlap closely in the principal component space (Figure 5), and cluster first with the smallest squared Euclidean distance (0.036) (Figure 6). Crucially, they share highly similar habitat conditions: comparable low altitudes (1372 m), high water temperatures (14.7°C–15.2°C), and the inferred moderate‐flow hydrological regime. This high morphological similarity despite geographical isolation is a classic hallmark of convergent evolution—whereby organisms from distinct ancestors or populations independently evolve analogous phenotypes in response to comparable environmental pressures (Technologies 2006).

Numerous classic examples of convergent evolution have been documented in fish. For instance, species of *Teleostei: Gobionellidae* distributed on the eastern and western coasts of the North Pacific, despite geographical isolation, have independently evolved similar body sizes and visual systems. Studies emphasize that microecological environments (e.g., light gradients)—rather than macrohabitat differences—drive this convergence (Ellingson et al. 2014). Similarly, the morphological convergence of the Karasu River and Toxkan River populations strongly suggests that the habitat template of “moderate flow velocity and relatively high water temperature” exerts strong directional selection pressure on the body structure of *D maculatus*. This pressure likely filters out phenotypes that deviate from the optimal state, thereby fostering consistency between the two populations in key functional traits such as body size and fin position (Reyes Corral and Aguirre 2019). While the possibility of historical gene flow cannot be entirely ruled out, the primary driver of such a high degree of morphological consistency is more likely parallel adaptation to similar ecological niches rather than shared ancestral history (Weber et al. 2021; Yang et al. 2025; Xu et al. 2019). The slightly longer snout of the Toxkan River population and the slightly wider mouth gape of the Karasu River population may indicate subtle microhabitat‐associated differentiation in their feeding strategies (Williams et al. 2017); however, this still requires verification in future research via direct dietary analysis (e.g., stomach content analysis or stable isotope techniques).

### 4.3 Evolutionary Mechanisms and Conservation Implications

The patterns of morphological clustering and discrimination identified in this study result from the combined effects of the evolutionary history and contemporary environmental pressures of *D maculatus*. The clustering of the Karasu River and Toxkan River populations provides strong evidence for convergent evolution. For the aggregation of the Kyzyl River and Muzati River populations, aside from partial similarities in environmental pressures, the potential influence of historical population expansion and gene flow events cannot be ruled out (Li et al. 2016). In contrast, the separation of the Kizil River population underscores geographical isolation (induced by reservoir construction) as a powerful driver of rapid population divergence—this divergence may involve changes in both phenotypic plasticity and genetic variation (Nicol et al. 2017; Li et al. 2020).

The 90.6% overall discriminant accuracy from discriminant analysis confirms that geographical populations of *D maculatus* can be effectively distinguished using morphological indices. Most of these key discriminatory traits (e.g., interorbital distance X_8_, caudal peduncle height X_12_) are functionally relevant, which supports the fundamental concept that “morphology is the carrier of ecological function” (McWhinnie et al. 2023; Shuai et al. 2018). This morphological–habitat mapping relationship allows morphological monitoring to act as a low‐cost and efficient indicator for assessing how environmental changes impact populations (Shuai et al. 2018; Maceda‐Veiga et al. 2014).

In conclusion, this study uncovers the spatial pattern of close associations between the morphological traits of *D maculatus* and key environmental factors (particularly temperature and habitat structure), emphasizing the pivotal role of environmental filtering in shaping population phenotypes. While the specific mechanisms underlying adaptive evolution (plasticity vs. genetic adaptation) require further elucidation, the results clearly demonstrate that different geographical populations are diverging along trajectories adapted to their local environments. This holds clear implications for the conservation of *D. maculatus*—an endangered species: populations across different river basins should be treated as management units with unique adaptive potential, and targeted conservation measures should be implemented. Notably, the Kizil River population—highly specialized due to human disturbances (e.g., reservoir isolation)—merits special attention given its unique evolutionary trajectory and potential vulnerability.

## 5 Conclusion

This study systematically analyzed the morphological differentiation pattern of five geographical populations of *D. maculatus* in the Tarim River Basin by integrating traditional morphology and geometric morphometrics with multiple statistical analysis methods, revealing the adaptive evolutionary mechanism of *D. maculatus* driven by the environment and providing new insights into the phenotypic differentiation of cold‐water fish. Follow‐up studies will combine genomics and eDNA technologies to further analyze the genetic basis of adaptive evolution and establish dynamic monitoring systems.

## Author Contributions

**Yichao Hao:** conceptualization (equal), data curation (lead), formal analysis (lead). **Huimin Hao:** conceptualization (supporting), data curation (supporting), formal analysis (supporting), methodology (supporting). **Zhengwei Wang:** conceptualization (supporting), data curation (supporting), formal analysis (supporting), methodology (supporting). **Yinsheng Chen:** resources (supporting). **Huale Lu:** project administration (supporting), resources (supporting). **Jie Wei:** project administration (supporting), resources (supporting). **Zhulan Nie:** conceptualization (lead), data curation (equal), formal analysis (equal), funding acquisition (lead), investigation (lead), methodology (equal), project administration (equal), resources (lead), supervision (lead), writing – original draft (equal), writing – review and editing (lead).

## Funding

This research was funded by the National Natural Science Foundation of China, 32460920; the Tarim University President's Fund Innovation Research Team Project, TDZKCX202204; Ministry of Science and Technology of the People's Republic of China, 2022xjkk150403. Science and Technology Bureau of the Production and Construction Corps (No. 2025YD016).

## Ethics Statement

All experimental protocols were approved by the Ethics Committee of Tarim University (approval code: PB20250627002; approval date: 27 June 2025) and complied with relevant laws, guidelines, and policies on animal welfare.

## Conflicts of Interest

The authors declare no conflicts of interest.

## Supporting information


**Data S1:** ece372616‐sup‐0001‐Supinfo.xlsx.

## Data Availability

Traditional morphological data and geometric morphometric data are provided in the Supporting Information.
